# Tear strength and elastic recovery of new generation hybrid elastomeric impression material: A comparative study

**DOI:** 10.1186/s13104-022-06110-3

**Published:** 2022-06-27

**Authors:** Lamia Singer, Christoph Bourauel, Shaymaa I. Habib, Heba El-Amin Shalaby, Sayed H. Saniour

**Affiliations:** 1 Oral Medicine Technology, Dental School, Medical Faculty, University of Bonn, 53111 Bonn, North Rhine-Westphalia Germany; 2 Dental Biomaterials Department, Faculty of Oral and Dental Medicine, Cairo University, Cairo, Egypt; 3 Dental Biomaterials Department, Faculty of Oral and Dental Medicine, British University in Egypt, Cairo, Egypt; 4 Dental Biomaterials Department, Faculty of Dentistry, October University for Modern Sciences and Arts, Cairo, Egypt

**Keywords:** Elastomers, Vinylsiloxenether, Tear strength, Elastic recovery, Polyvinylsiloxane, Polyether

## Abstract

**Objective:**

Since there is no material in the market met all the ideal requirements of an impression material, thus in an attempt to find one, hybridization between the two most commonly used impression materials were done. The aim of the hybridization was to obtain a new material combining the good merits of both and eliminate their shortcomings. Thus, this study aimed to assess the impact of hybridization between polyether with addition silicone on tear strength and elastic recovery of the new material and compare such effect with regard to parent materials.

**Results:**

A polyether (PE), polyvinyl siloxanse (PVS) and vinyl polyether silicone (VPES) hybrid elastomers were used in the present study. Tear strength was measured one hour after setting time of each material according to the manufacturer and the three materials showed statistically comparable tear strength in N/mm. Elastic recovery was evaluated one minute after the setting time recommended by the manufacturer. The three materials were statistically insignificant from each other, and all met the ISO4823 requirement of having greater than 96.5% recovery.

## Introduction

Polyvinylsiloxanes (PVS) and polyethers (PE) are the most commonly used rubber base materials for secondary impressions all over the world [1]. Polyethers have excellent flow and hydrophilic properties, but they are rigid, with slow recovery and low tear strength. On the other hand, PVS has great stability, high tear strength, and excellent recovery, but with a hydrophobic nature [2]. As there is no single impression material meets all of the ideal requirements, significant improvements have been made all over the years, including the introduction of vinyl polyether silicone (VPES) hybrids. VPES were introduced to represents the blend of hydrophilicity of polyether and excellent elastic recovery as well as the dimensional stability of Polyvinylsiloxanes impression materials [3].

The tear of elastomeric materials is commonly initiated and propagated at high stress concentration sites as defects, or deformations within the material. [4]. From the clinical perspective, materials with high tear energy or tear strength are not necessarily considered to be superior to the materials with low tear energy or tear strength [5]. Ideally, impression material should absorb high energy prior to permanent deformation and tears rather than deforms at critical areas. Tear propagation energy have been employed as common In vitro method to evaluate tear strength of elastic dental materials. [6]. Polyvinylsiloxanes have the ability to absorb over three times more energy up to the point of permanent deformation than other elastomers [7].

The accuracy of dental elastomeric impression materials greatly depends on their ability to recover elastically after a deformation; the greater the recovery, the better the precision [8]. Strain in compression represents the flexibility or stiffness of an impression material [9]. The lower the strain in compression, the stiffer the material, this factor indicates whether the polymerized impression can be removed from the oral cavity without injury to the oral tissues. Elastic recovery also indicates whether the set material will resist deformation when a gypsum product is poured into it and whether the set gypsum material can be removed easily and safely from the impression [10]. Lu et al. [9]**,** found that strain in compression was inversely correlated to elastic recovery, the higher the elastic recovery, the lower the strain in compression.

It is still debatable whether the hybridized material surpassed the properties of the parent ones and only few studies compared them all. The purpose of this study was to evaluate the tear strength, and elastic recovery of two of the generally used elastomeric impression materials (PVS and PE), and to compare them with the last launched material produced from their hybridization (VPES). This study also aimed to solve the conflict between the different studies published by competitive manufacturers.

## Main text

### Materials

Three commercially available elastomeric impression materials (Table 1) were used in this study.

**Table 1:** Materials used in the study

Impression Materials	Vinyl polyether silicone hybrid	Polyether	Addition Silicone
Commercial name	Identium Medium	Impregum Penta Medium	Express XT Medium
Manufacturer	KETTENBACH Germany	3 M ESPE St. Paul, USA	3 M ESPE Germany
Working time (min:sec)	2:00	2:45	1:30
Setting time (min:sec)	5:30	3:15	2:30
Mixing device	Pentamix 3	Pentamix 3	Garant dispenser

### Methods

#### Tear strength

Specimens of each material were prepared using a mold recommended by American society for testing materials (ASTM) specification for tear strength “Die C 12”. The mold dimensions were 96.4 mm length, 19.5 mm width and 13.7 mm thickness at the tearing point.

The mold was placed on a flat glass slab and was filled with the material to be tested, another glass slab was placed on the top of the filled mold and a 500 g weight was placed on top of that second glass slab until setting occurred. A universal testing machine (Model LRX—plus: Lloyd, Farcham, UK) equipped with a custom-made grip was used to induce tensile strain at a rate of 50 mm/min until rupture [11, 12]. The load at rupture was used to determine the tear strength according to the following equation: T = F\d Where: T: Tear strength in N\mm, F: Tearing force, d: Thickness of the specimens.

#### Elastic recovery

Specimens were made for each material to evaluate the elastic recovery according to ISO 4823 [13]. Specimens were made using a mold formed of a fixation ring (20.5 mm inside diameter, 19 mm height) and a split plastic mold (12.5 mm inside diameter, 20.5 mm outside and 20 mm height). Each material was mixed, placed inside the mold and a glass plate was pressed on the top to remove the excess and to form a flat smooth surface of the specimen. The assembly was immersed in a water bath (32 + 1 °C) until the end of the known initial setting time of each material.

Elastic recovery was evaluated 60 s after the initial setting time [14, 15]. Each specimen was placed on dial indicator (Mitutoyo Europe GmbH, Neuss, Germany), and the spindle was lowered for 10 s, the initial reading was recorded as reading (A) in mm. Five second later, the specimen was deformed using a universal testing machine to a height of 16 mm (20% strain) within 4 s and the deformation was maintained for 5 s and then released. Thirty seconds after the release, the specimen was placed again on the dial indicator and reading B in mm was recoded. The percentage of compression set was calculated from the given equation as an average of three determinations and was recorded to the nearest 0.1%

$$\% {\text{ Compression set}} = \, \left( {{\text{A}} - {\text{B}}} \right)/{\text{ A }} \times { 1}00)$$

#### Statistical analysis

Numerical data were presented as means and standard deviation (SD) values. All quantitative variables showed parametric distribution thus, One-way Analysis of Variance (ANOVA) was used for comparison between the groups. Tukey’s post-hoc test was used for pair-wise comparison when ANOVA test was significant. Chi-square (× 2) test was used to compare between the groups. The significance level was set at P ≤ 0.05. Statistical analysis was performed using SPSS (SPSS, Inc., an IBM Company, USA) Statistics Version 20 for Windows.

### Results

#### Tear strength

**Fig. 1:**
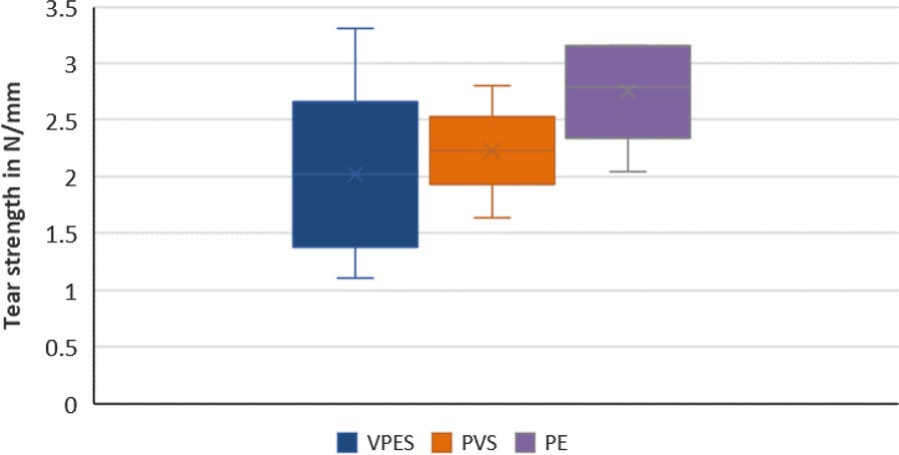
Box plot chart representing the tear strength of the three tested impression materials

The variables showed parametric distribution, and thus one way ANOVA was used followed by Tukey’s post hoc for pairwise comparison between the tested groups to evaluate the tear strength. Statistical analysis of the means of the tear strength (in N/mm) for the three tested materials are illustrated in Fig. 1. Results revealed that there was statistically insignificant difference between tear strength of the three materials (*P*-value = 0.167). PVS showed mean tear strength of 2.23 (SD = 0.41) N/mm, whereas each of PE and PVES hybrid recorded mean tear strength values of 2.76 (SD = 0.46) N/mm and 2.02 (SD = 0.81) N/mm respectively.

#### Elastic recovery

**Fig. 2:**
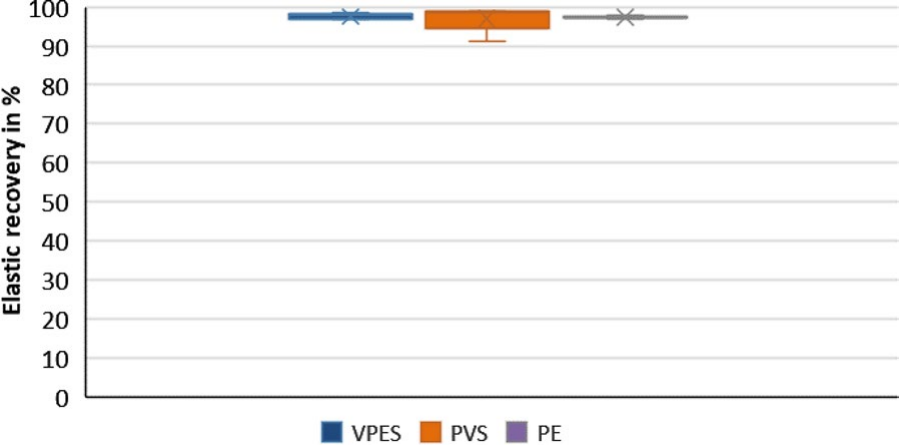
Box plot representing the elastic recovery in the three tested impression materials

Statistical analysis of the means of recovery from deformation (in percentage) for the three tested impression materials are represented in Fig. 2. Results indicated that there was no statistically significant difference in the elastic recovery of the three materials (*P*-value = 0.908). PVS recorded a mean elastic recovery of 98.5% (SD = 3.35), while VPES hybrid and PE showed mean values of 97.5% (SD = 0.79) and 97.2% (0.4) respectively. All of the materials tested met the ISO4823 requirement of having greater than 96.5% recovery.

### Discussion

#### Tear strength

Setting time of the impression material is strongly correlated to its tear strength. Shorter setting times are more convenient for clinicians and patients, but if the setting time is inadequate and the impression material has not completely polymerized before removal, the impression material will tear [16].

In the present study, tear strength test was performed one hour after the setting time of each material described by the manufacturer. Although there is no statically significant difference between the three materials, it was found that the tear strength of polyether was higher than that of polyvinylsiloxane or vinyl polyether silicone hybrids. This was in agreement with the studies carried out by Huettig et al. [17], but inconsistent with results obtained by Lawson NC et al. [16], who stated that addition silicone materials provided higher tear strength than polyether materials. This could be due to usage of different consistencies and tearing rates compared to the present study.

The relative level of hydrophilicity or hydrophobicity of the different materials may affect the interactions between the impression materials and sulcus fluids. The incorporation of these fluids during polymerization could result in defects that act as stress initiators, reducing the tear strength of the polymerized material [18]. On the other hand, polyvinylsiloxanes deform at much slower rates and tear at points of less permanent deformation than do the other elastomeric impression materials [18], and they are less rigid than polyether when set. Tear strength can vary with different products, film thicknesses and varying temperature performed to adjust the working time [19].

The vinyl polyether silicone hybrids material exhibited a slightly lower tear strength compared to polyvinylsiloxane. Whether this relative lower tear strength can cause tear of impression material intraorally, or on separation from casts requires further investigation. Moreover, it is worth mentioning that the adhesion of the material to the teeth and soft tissues, and the presence of internal voids, which greatly affect tear strength, have not been assessed in the present study [20].

#### Elastic recovery

Impression materials are polymers with highly flexible kinked segments that uncoil and move freely on loading. Upon removal of the load, an ideal elastomer will exhibit complete elastic recovery and return to its pre-stressed configuration. The degree to which this occurs is a measure of the elastic recovery of the material [21]. The degree of cross-linking of the polymer chains, temperature, and the rate of applied strain greatly affect the amount of permanent deformation of the material [18].

In the present study, elastic recovery was tested by compression set rather than tension. All of the materials in this study met the requirement of ISO 4823, which requires greater than 96.5% elastic recovery. The results showed that the mean elastic recovery of polyvinylsiloxane was (98.5%) followed by vinyl polyether silicone hybrid (97.2%) and then Polyether (97.5%), although the differences may not be clinically significant.

For polyvinylsiloxane materials, the elastic recovery is dependent on the components, such as base silica, copolymer filler, and chain extenders [22]. Polyvinylsiloxanes have the least viscoelastic qualities thus requiring the least time for recovery from viscoelastic deformation [18]. A study reported that polyvinylsiloxanes have sufficient elastic recovery to allow an impression to be poured only six minutes after removal from the mouth [23]. These results contradict Lu H et al. [9], who stated that polyether had a higher elastic recovery compared to the new additional silicone materials. This might be due to the difference between the materials brands, viscosities and methodology used in the present study.

Vinylpolyether silicone hybrid material was ranked between Polyether and Polyvinylsiloxane; since it is a hybrid material containing polyether and siloxane groups’, polyether material yield less elastic recovery than polysiloxane materials, therefore, it is elastic recovery lies in between them. This is in agreement with Lawson et al. [16].

### Conclusions

The hybrid VPES material has showed in this study elastic recovery and tear strength values comparable with the parent materials (PE and PVS). Therefore, the hybridization did not seem to affect those two properties negatively, nevertheless verification of enhancement of other material properties should be assessed as well with newly available techniques.

## Limitations

One limitation of the elastic recovery testing in the present study is that recovery from tensile strain had not been evaluated. Mansfield M and Wilson H [24] performed a study comparing recovery from 50% tensile strain and compressive strain, concluding that both tests were necessary. However, Blomberg et al. [23] found a strong correlation between elastic recovery from tensile and compressive strain and concluded that only one method is required.

## Data Availability

The datasets used and/or analysed during the current study are available from the corresponding author on reasonable request.

## Abbreviations

PE: Polyether
PVS: Polyvinylsiloxanes
VPES: Vinyl polyether silicone
ANOVA: One-way Analysis of Variance
